# Extrathyroidal extension and cervical node infiltration are associated with recurrences and shorter recurrence-free survival in differentiated thyroid cancer: a cohort study

**DOI:** 10.1186/s13044-022-00131-7

**Published:** 2022-07-26

**Authors:** Sumadi Lukman Anwar, Roby Cahyono, Suwardjo Suwardjo, Herjuna Hardiyanto

**Affiliations:** Department of Surgery, Division of Surgical Oncology, Dr. Sardjito Hospital / Faculty of Medicine, Public Health and Nursing, Universitas Gadjah Mada, Jl Kesehatan No. 1, Yogyakarta, 55281 Indonesia

**Keywords:** Differentiated thyroid cancer, Recurrence, Risk factors, Extrathyroidal extension

## Abstract

**Background:**

Differentiated thyroid cancer has excellent overall survival. However, around 20% of patients experience recurrent diseases after a certain time of follow-up. Therefore, identification of risk factors for recurrence is necessary to adjust treatment and surveillance planning.

**Methods:**

A retrospective study was conducted of 312 patients with differentiated thyroid cancer who received surgery with and without adjuvant treatment. Clinical and pathological risk factors were analyzed for recurrences.

**Results:**

After median follow-up of 57 months, 109 of 312 patients (34.9%) developed recurrences. Extrathyroidal extension and positive cervical nodes were significantly associated with recurrences (OR = 2.449, 95%CI:1.260–4.760, *P* = 0.008 and OR = 3.511, 95%CI:1.860–6.626, *P* < 0.001; respectively). Lympho-vascular invasion (LVI) and tumor multifocality were also associated with increased risk of recurrence (OR = 2.577, 95%CI:1.380–4.812, *P* = 0.003 and OR = 1.602, 95%CI:1.001–2.495, *P* = 0.050; respectively). Using multivariable regression, only older age and tumor infiltration to the lymph nodes were significantly associated with recurrences (OR = 2.227, 95%CI:1.037–4.782, *P* = 0.040 and OR = 2.966, 95%CI:1.470–5.986, *P* = 0.002; respectively). In addition, T4, cervical lymph node infiltration, older age, and LVI were associated with shorter recurrence-free survival.

**Conclusion:**

Recurrence rates in our study population are relatively high. Extrathyroidal extension, positive neck lymph node, and older age were associated with recurrence risks of well differentiated thyroid cancers.

## Background

Thyroid cancer is the most frequently diagnosed endocrine-related cancer and the incidence has sharply increased in the past three decades. More than 500,000 thyroid cancers were diagnosed in 2020 worldwide and 13,000 cases were from Indonesia [1]. Approximately 90% of thyroid cancers are differentiated papillary and follicular adenocarcinomas [2, 3], which have excellent survival with more than 95% of patients surviving after 10 years follow-up [2, 3]. However, around 20% of patients develop locoregional and distant recurrences during the course of their lives after the treatment [2, 4].

The rapid increasing incidence of thyroid cancer in developed nations has been associated with the increased imaging to detect early thyroid nodules. Incidental findings through advanced imaging has also been suggested to contribute to overdiagnosis and overtreatment [5]. However, the increasing incidence of thyroid cancer in Indonesia is also accompanied with the increasing mortality during the past two decades [6, 7]. Around 2,200 deaths were associated with thyroid cancer in Indonesia in 2020 [6, 8, 9]. The increasing mortality rates might be associated with more advanced stages at diagnosis and lack of treatment facilities. However, older age and metabolic comorbidity including hypertension, dyslipidemia, and cardiovascular diseases have also been associated with an increased risk of mortality among thyroid cancer survivors. Cancers in low and middle-income countries are usually diagnosed in later stages hindering the patients’ prognosis and their quality of life [9, 10]. Weak health system, poor accessibility to healthcare providers, and lower awareness have been associated with the delayed timing of diagnosis and treatment which might also affect the long-term prognosis [9–11].

Well differentiated thyroid cancer is a highly curable disease with 5-year-survival rates above 95% [12, 13]. Initial diagnosis, surgery, thyroid ablation, and suppression therapy have substantial impacts in lowering locoregional recurrences [13]. However, around 15% of patients require salvage treatment because of the uncontrolled locoregional recurrence and distant spread [13, 14]. In patients with recurrence, the mortality increases into 38-69% [14]. Identification of variables associated with recurrences is very important to avoid underestimation and inadequate treatment particularly for patients diagnosed in late stages. Studies addressing patterns of recurrences and risk assessment of thyroid cancer are still lacking in low-income countries. In addition, identification of individuals with higher risks of recurrence will help the clinicians to design surveillance and disease monitoring plans. Accordingly, using retrospectively collected data from a patient cohort, this study aimed to identify determinants associated with risks of recurrence in differentiated thyroid cancers.

## Material and methods

### Study subjects

Attributable baseline pathological and demographic variables of patients with thyroid cancer diagnosed at the Department of Surgery at our hospital were accumulatively collected. All patients received standard treatment according to the national guidelines for differentiated thyroid cancer. Age at diagnosis was stratified according to the recurrence risks of thyroid cancer [15]. Tumor size and lymph node infiltration were categorized according to the 8^th^ Edition of the American Joint Committee on Cancer (AJCC) [13]. Tumor focality, lympho-vascular invasion (LVI), extrathyroidal extension (ETE), and margin status were determined according to the pathology reports and were classified into dichotomous variables. Follow-up and surveillance were accomplished according to the institutional guidelines for patients with thyroid cancer. This cohort study was conducted following the ethical guidance according to the 1964 Declaration of Helsinki. The study has been approved by our hospital’s Medical and Health Research Ethics Committee (0471/EC/2021).

### Recurrence-free survival (RFS)

Recurrence was determined as re-emergence of differentiated thyroid cancer at the thyroid bed, lymph node, or distant metastasis at least 6 months after disease-free status that was confirmed with imaging and pathological report. Disease-free was defined as no evidence of residual disease, local recurrence, nor distant metastasis through physical examination and respective imaging, non-stimulated thyroglobulin below 1 ug/L, or negative uptake of radioiodine scan. Recurrence-free survival (RFS) was calculated for each patient from the first pathological report to the time point of recurrence or the last follow-up.

Lobectomy (isthmusectomy and subtotal thyroidectomy) was performed for patients with low risk according to the American Thyroid Association (ATA) guidelines. Total thyroidectomy with or without radionucleotide ablation was performed for patients with intermediate and high risk. Additional modified neck dissection was performed for patients with lymph node involvement. Central lymph node dissection is not routinely performed in our medical center. To facilitate statistical analysis, we classified surgery into lobectomy and total thyroidectomy.

### Statistical analysis

Baseline clinical and pathological variables were compared and summarized in frequency tables. The association of clinical characteristics was analyzed with univariable and multivariable logistic regression using recurrence as a dependent variable and was presented with Odds ratio (OR), 95% confidence intervals (CI) and *P-values*. All statistical tests were completed using SPSS 17.0 software (IBM Corp., Chicago, IL).

## Results

### Baseline characteristics of differentiated thyroid cancer

Among 312 patients with differentiated thyroid cancers, 183 (58.7%) were over 45 years old, 254 (81.4%) were females, and 290 (92.9%) were Javanese ethnicity. Median age at diagnosis was 48 years and mean tumor size was 6.4 cm. There were 251 (80.4%) patients who had tumors with diameter larger than 4 cm and 48 (15.4%) who had positive neck lymph nodes. There were 48 (15.4%) patients reported with LVI and 41 (13.1%) patients with ETE. There were 226 (27.4%) patients who underwent total thyroidectomy and 136 (56.4%) patients who received radionucleotide ablation at least once (Table 1).

**Table 1:** Baseline characteristics of differentiated thyroid cancer patients (*N* = 312)

Baseline characteristics	N (%)
Age	
< 45 years	129 (41.3%)
45–65 years	150 (48.1%)
> 65 years	33 (10.6%)
Sex	
Female	254 (81.4%)
Male	58 (18.6%)
Ethnicity	
Javanese	290 (92.9%)
Non-Javanese	22 (7.1%)
Tumor size (T)	
T1	6 (1.9%)
T2	55 (17.6%)
T3	212 (67.9%)
T4	39 (12.5%)
Nodal status	
Negative	264 (84.6%)
Positive	48 (15.4%)
Stage	
1	224 (71.8%)
2	63 (20.2%)
3	25 (8.0%)
Presence of extrathyroidal extension	
Yes	41 (13.1%)
No	271 (86.9%)
Number of lesion	
Unifocal	185 (59.3%)
Multifocal	127 (40.7%)
Lymphatic invasion	
Negative	264 (84.6%)
Positive	48 (15.4%)
Surgery	
Total thyroidectomy	226 (72.4%)
Lobectomy	86 (27.6%)
Radionucleotide ablation	
Yes	136 (43.6%)
No	176 (56.4%)

### Factors associated with risk of recurrence in differentiated thyroid cancers

After median follow-up of 57 months, we observed 109 (34.9%) patients experienced recurrent diseases. According to the stages, recurrence rates were 29%, 43%, and 68% in Stage I, Stage II, and Stage III; respectively (Table 2). Using univariable logistic regression, we found that male and older age at diagnosis were associated with higher risks of recurrence (OR=1.993, 95%CI:1.118-3.556, *P*=0.019 and OR=2.850, 95%CI:1.367-5.941, *P*=0.005; respectively). Among the patients with differentiated thyroid cancer, ETE and involvement of neck lymph nodes were also significantly associated with higher risks of recurrence (OR=2.449, 95%CI:1.260-4.760, *P*=0.008 and OR=3.511, 95%CI:1.860-6.626, *P*<0.001; respectively), (Table 3). LVI and tumor multifocality were also associated with higher risk of recurrence (OR=2.577, 95%CI:1.380-4.812, *P*=0.003 and OR=1.602, 95%CI:1.001-2.495, *P*=0.050; respectively). In general, patients who had total thyroidectomy were associated with lower rates of recurrence (OR=0.393, 95%CI:0.219-0.704, *P*=0.002) and there was no significant association with recurrence among those who received radionucleotide ablation (OR=1.153, 95%CI:0.722-1.842, *P*=0.552; respectively), (Table 3). In the multivariable regression analyses, only older age and tumor infiltration to the lymph nodes were associated with higher risks of recurrence (OR=2.227, 95%CI:1.037-4.782, *P*=0.040 and OR=2.966, 95%CI:1.470-5.986, *P*=0.002; respectively)

**Table 2:** Distribution of recurrence rates across baseline TNM stages

Stage	Recurrence rates
Stage I	65/224 (29.0%)
Stage II	27/63 (42.8%)
Stage III	17/25 (68%)

**Table 3:** Odds ratios and 95% confidence intervals of recurrence risks in differentiated thyroid cancer patients using univariable and multivariable logistic regression

Variable	Category	Univariable analysis		Multivariable analysis	
		OR (95%CI)	*P*-value	OR (95%CI)	*P*-value
Age	≥ 65 years	2.850 (1.367–5.941)	**0.005**	2.227 (1.037–4.782)	**0.040**
	< 65 years	Ref		Ref	
Sex	Male	1.993 (1.118–3.556)	**0.019**	1.818 (0.961–3.438)	0.066
	Female	Ref		Ref	
Ethnicity	Javanese	1.901 (0.681–5.291)	0.220	1.845 (0.614–5.556)	0.275
	Non-Javanese	Ref		Ref	
Tumor size	T4	1.932 (0.982–3.800)	0.057	1.937 (0.838–4.479)	0.122
	T1T2T3	Ref		Ref	
	T3	0.878 (0.479–1.612)	0.676	1.614 (0.642–4.058)	0.308
	T1T2	Ref		Ref	
Extrathyroidal extension	Yes	2.449 (1.260–4.760)	**0.008**	2.431 (0.525–11-254)	0.256
	No	Ref		Ref	
Node status	Positive	3.511 (1.860–6.626)	** < 0.0001**	2.966 (1.470–5.986)	**0.002**
	Negative	Ref		Ref	
Histology	Papillary	1.984(0.993–3.968)	0.053	2.134 (0.378–12.044)	0.391
	Follicular	Ref		Ref	
LVI	Positive	2.577 (1.380–4.812)	**0.003**	1.417 (0.706–2.844)	0.340
	Negative	Ref		Ref	
Multifocal tumor	Multifocal	1.602 (1.001–2.495)	**0.050**	1.211 (0.719–2.041)	0.472
	Unifocal	Ref		Ref	
Surgery	Total thyroidectomy	0.393 (0.219–0.704)	**0.002**	0.627 (0.323–1.220)	0.169
	Lobectomy	Ref		Ref	
RAI	Yes	1.153 (0.722–1.842)	0.552	1.290 (0.256–2.431)	0.582
	No	Ref		Ref	

*T* Tumor size

*LVI* Lympho-vascular invasion

*RAI* Radioactive iodin ablation

### Recurrence-free survival (RFS)

**Fig. 1:**
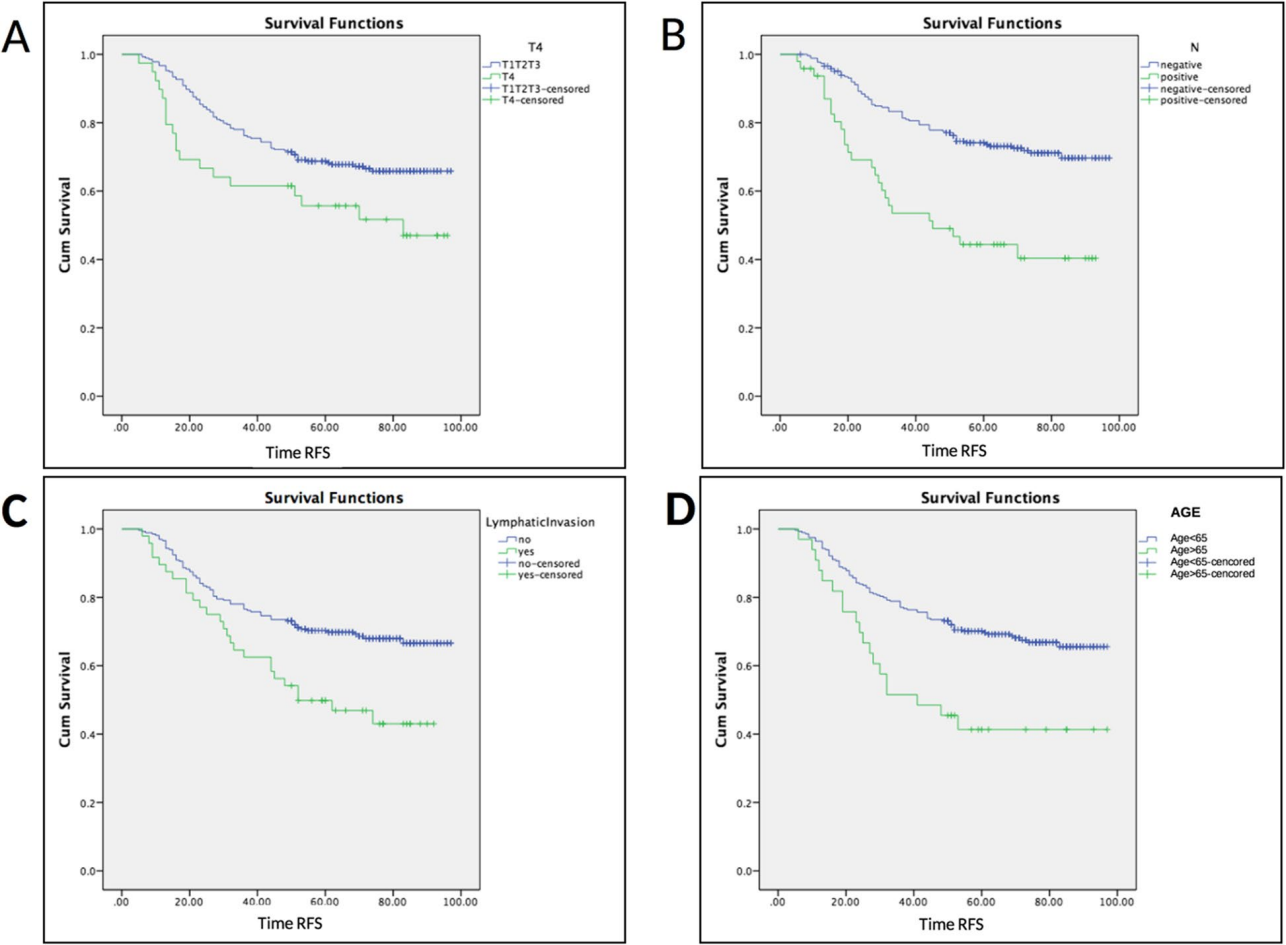
Kaplan–Meier recurrence-free survival curves showing a significantly lower time to recurrences in patients with T4 (mean RFSs were 60.8 vs 74.4 months, Log-Rank test *P* = 0.022, panel **A**), positive cervical lymph nodes (Mean RFSs were 53.9 vs 78.3 months, Log-Rank test *P* < 0.001, Panel **B**), positive lympho-vascular invasion (Mean RFSs were 52.3 vs 74.9 months, Log-Rank test *P* = 0.015, Panel **C**), and older age more than 65 years (Mean RFSs were 54.9 vs 74.8 months, Log-Rank test *P* = 0.001, Panel **D**)

Using Kaplan-Meier curve, patients’ RFS status were compared according to the clinicopathological variables. Patients with ETE to the nearby tissues (T4) had significantly shorter RFS compared to those with tumors within the thyroid bed (Mean RFSs were 60.8 vs 74.4 months, Log-Rank test *P*=0.022), (Fig. 1). Involvement of neck lymph nodes was also associated with poorer RFS compared to node negative patients (Mean RFSs were 53.9 vs 78.3 months, Log-Rank test *P*<0.001). Positive LVI was also associated with shorter RFS (Mean RFSs were 57.9 vs 75 months, Log-Rank test *P*=0.002). In our study cohort, male patients and age older than 65 years were also associated with shorter RFS (Mean RFSs were 52.3 vs 74.9 months, Log-Rank test *P*=0.015 and 54.9 vs 74.8 months, Log-Rank test *P*=0.001; respectively).

## Discussion

Patients with differentiated thyroid cancer generally have good prognosis and undergo life-time follow-up or suppressive treatment. However, recurrence rates are around 20% and more than one third occur after five years of initial treatment [16]. Recurrence rates and clinical-pathological variables associated with thyroid cancer recurrence in low- and middle-income countries are still under-reported [10]. Identification of factors attributed to disease recurrence is very important to provide cost-effective management and surveillance schemes [14, 16]. Although remarkable improvement has been made during the past two decades for treatment and detection of postoperative recurrences, the clinical recommendations have not been implemented in every hospital. Especially in low-resource settings, there are typically only a few centers that have facilities for thyroid scan, radionucleotide ablation iodine (RAI) testing, and systematic monitoring and follow-up [10, 17].

In our patient cohort, recurrence rates were relatively high reaching 29%, 43%, and 68% for patients initially diagnosed in Stage I, Stage II, and Stage III; respectively (Table 2). Gan et al. [18] reported a recurrence rate of 3% among patients with differentiated thyroid cancers after a median follow-up of 27 months. A recent study found significantly different recurrence rates in patients diagnosed in early and advance stages (7.2% vs 28.2%) [19]. Liu et al. reported a higher loco-regional recurrence in patients with positive lymph nodes (31.5%) in comparison to those with node negative (5.2%) [20]. In comparison to these reports, our study showed relatively higher recurrence rates including in patients initially diagnosed in Stages I and II.

We identified several variables associated with increased risk of recurrences including older age, being male patients, primary thyroid cancer with ETE, and positive neck lymph nodes. In the multivariable analysis, however, only older age and positive lymph nodes were significantly associated with disease recurrence (Table 3). Hollenbeak et al. reported recurrence rates up to 39% in older patients with differentiated thyroid cancer [21]. Thyroid cancers are generally diagnosed in patients younger than 55 years and around 20% are initially detected in individuals above 65 year-old [2, 21]. In our cohort, only 10% patients were diagnosed above 65 years (Table 1); yet, they were associated with higher risks of recurrence (Table 3). Females are more affected by goiter and differentiated thyroid cancer, although the etiology is still not fully addressed. However, males affected by differentiated thyroid cancer are associated with poor prognosis [22, 23]. Other studies, on the other hand, have shown that sex is not an independent risk factor for poor prognosis [24, 25]. Male individuals with thyroid cancer more frequently present in advanced stages, with larger tumor size, positive lymph nodes, and ETE [22, 26], which might also explain the association with higher risks of recurrence. Sequential analysis shows that adverse outcomes attributable to the male sex gradually decrease over time with the improvement of diagnosis and treatment [27].

Our study showed the significant association of positive lymph nodes with higher recurrence rates. Involvement of neck lymph nodes has been associated with higher rates of thyroid cancer recurrence and mortality [20, 28], although the strength of the association varies widely among studies. With the improvements to detect involvement of lymph nodes, the indication for neck dissection in differentiated thyroid cancer is still debatable, particularly for micrometastasis to the lymph nodes. Rather than simply classify patients into binary variables (positive and negative), Schneider et al. found that the lymph node ratio was also associated with papillary thyroid cancer recurrence [29]. Collinearity should also be considered as patients with positive lymph nodes tend to have other clinicopathological adverse features including larger tumor size, ETE and LVI. Our data also showed that ETE, LVI and multifocal tumors were significantly associated with higher recurrence rates. ETE is considered as tumor extension outside the thyroid capsule with infiltration into surrounding tissues including strep muscles, trachea, larynx, laryngeal nerve, jugular vein and carotid artery. In our cohort, 13% patients had ETE (Table 1), which was parallel with previous studies (9-12%) [30]. According to ATA guidelines, the presence of ETE is considered with intermediate risk factors and extensive ETE is associated with high risk and both are associated with an increased risk of recurrence [13]. LVI is also associated with worse prognosis in differentiated thyroid cancer [31]. Our study found patients with LVI had a higher risk of recurrence, although it was not an independent factor. Wagner et al. have also revealed that LVI is associated with worse RFS especially in the presence of positive lymph node infiltration [31].

We also found that T4, positive neck lymph nodes, older age, and LVI were associated with shorter RFS (Fig. 1). Palme et al. [14] also reported that ETE (T4) and advanced stages at initial diagnosis were the most significant independent risk factors of recurrence in patients with well-differentiated thyroid cancers. Using the National Cancer Institute’s Surveillance, Epidemiology, and End Results (SEER) database, Banerjee et al. [32] revealed that tumor size, stage at diagnosis, and receiving radioactive iodine were associated with higher risks of recurrence in patients with differentiated thyroid cancer. In addition to higher frequency of ETE, our cohort also showed much larger tumor size (mean 6.4 cm) in comparison to other studies (mean tumor sizes were 2.1 cm [30] and 3.3 cm [32]). Parallel with our findings, Ito et al. [33] showed that older age was a prominent prognostic factor for overall survival in comparison to positive node and metastatic lesions in patients with papillary thyroid cancer. Another report demonstrated that primary tumor size at diagnosis and cervical nodal status were associated with shorter disease-free survival in patients with differentiated thyroid cancer [34].

Our study highlights some risk factors of disease recurrence among patients who are diagnosed with relatively larger tumor size (mean 6.4 cm) and advanced stages of differentiated thyroid cancer. The relatively higher rates of recurrence rates particularly in those who are initially diagnosed in Stages II and III within a median follow-up of 57 months might indicate that improvements of diagnosis, clinical management, and surveillance are warranted. In general, differentiated thyroid cancer has very good prognosis and recurrence usually occurs after a period of follow-up of more than 5 years [12, 35]. In our cohort, more than 80% of the patients were diagnosed with tumor size larger than 4 cm (Table 1) indicating that public awareness concerning thyroid cancer might need to be improved. Referral and health care system, assessment, and diagnosis of thyroid lesions should be improved to reduce delayed detection and treatment. Implementation of risk assessment, safe surgery procedures, and expansion of adjuvant RAI also need to be improved to provide more comprehensive treatment for patients with differentiated thyroid cancer in our region. However, there are some limitations of this study including factors associated with the single center, retrospective study design, and duration of follow-up and surveillance. Future multicenter studies with more comprehensive assessment of clinical markers, metabolic comorbidities, as well as social determinants are required to inform and improve the management of differentiated thyroid cancer and to reduce the recurrence rates.

## Conclusions

We highlight that recurrence rates of differentiated thyroid cancers in our study population are relatively high. Several variables at diagnosis including extrathyroidal extension, positive neck lymph node, and older age are significantly associated with higher recurrence risks and shorter recurrence-free survivals. Further research involving more patients on a regional and national scale is required to identify recurrence rates and the associated risk factors as well as to improve early detection, and to deliver better treatment and surveillance.

## Data Availability

The dataset is available upon reasonable request to the corresponding author.

## Abbreviations

AJCC: American Joint Committee on Cancer
ATA: American Thyroid Association
ETE: Extrathyroidal extension
CI: Confidence Interval
RFS: Recurrence-free survival
LVI: Lympho-vascular invasion
OR: Odds Ratio
RAI: Radionucleotide ablation iodine
T4: Any size of tumor with extrathyroidal extension
